# Living with Dysphagia

**DOI:** 10.3390/geriatrics7020040

**Published:** 2022-03-29

**Authors:** John Mirams

**Affiliations:** Chichester PO18 8QF, UK; john.educationrbe@outlook.com

I am 88 years old—a widower and retired businessman living in West Sussex.

I cannot remember ever going to the doctor in my first 25 years, but in 1959, life radically changed when I was diagnosed with throat cancer (Lymphosarcoma in the nasopharynx). I was treated daily for some weeks in St Thomas’s Hospital, London, with deep X-ray therapy and with cobalt rays to both sides of my head. My consultant, Mr Fleming, was magnificent, and thankfully, contrary to the original prognosis, the results were stated to be ‘remarkable’. I returned to work, and in due course, qualified for normal life insurance.

I am not sure about the timing or the extent of the damage to the bone cells in my jaw and head (osteoradionecrosis) that resulted from my cancer treatment, but over the years, I first lost all my teeth, then my hearing, and more recently, in 2009, my balance. 

In examining the balance problem, ENT revealed that I had developed a hole from the left ear canal into the mastoid bone (causing daily discharge) and a hole through the ear drum, requiring regular examination and aural cleaning. This they did every few months for nine years, and also gave me 40 sessions of hyperbaric treatment.

Apart from all this, I have kept the NHS quite busy in requiring operations for a hernia, for prostate problems, for two eye cataracts and for skin cancer—and their service and support have been wonderful throughout.

I think it was about the year 2000 when I began to irritate my wife and family by constantly coughing and clearing my throat during meals and snacks—but at first, it was assumed by some of them that it was just an irritating habit of developing old age!

The coughing became worse, and in 2005 I talked to my GP, who arranged a ‘Barium Swallow’ check for me. This showed that I did have some swallowing problems (or in medical terms ‘dysphagia’), but there was ‘no serious cause’ for concern, and that I should be OK, if I ‘took care when eating, and took plenty of fluids’.

However, the swallowing became more difficult, mainly due, I thought, to the huge quantities of saliva which I produced every day, and which had been described by one St Thomas’s consultant as being ‘like old rope’.

I changed the foods I ate, avoiding most of the ‘High Risk’ foods, as listed on the Web—especially vegetable and fruit skins, most tough meats, most fresh breads, most crisps, all nuts, dried fruits, many cereals, mueslis and many biscuits.

Despite this change, mealtimes continued to be an ordeal, involving regular choking fits, which normally required a visit to the bathroom, lasting perhaps 5 or 10 min; food or drink being discharged through the nose; and coughing, choking or sneezing causing minor nosebleeds, mainly from the right nostril only, or sometimes a full-scale bleed.

Having a full set of dentures became an increasing hazard, with a tendency for some foods to cling tightly to the upper palate and with the lower set being dislodged. Ever since, I have had a real fear of swallowing the lower denture completely, during a major coughing or choking episode.

Social activity was obviously hazardous, and matters came to a head in 2012 during a smart club dinner with some 40 other guests, when I choked and deposited a mouthful of food and drink, plus a lot of blood, over one of the tables and two unfortunate diners!

Apart from such dramatic occasions, the whole process of eating had become a tiresome and boring activity, and as a result I lost about a stone in weight.

The impact of dysphagia on my social life after the age of 64 was substantial. I avoided all possible social events that might involve eating or drinking, and for example in the early stages I opted out of invitations to old school and college functions, to club dinners and to any business meetings and contacts with customers, etc., which might involve refreshments.

Card evenings, dinners with old friends, family gatherings, birthdays and even Christmas celebrations were always a time of tension and potential embarrassment, from having to refuse certain dishes and remembering to say ‘No’ to offers of my favourite caramel chocolates!

After my wife died and after rashly attending the disastrous club dinner referred to above, my social life totally collapsed, and I therefore rapidly adopted a semi-reclusive lifestyle, concentrating on research and writing about matters of national interest.

Therefore, although dysphagia has been an inconvenient, irritating and sometimes very embarrassing feature of life, especially in the last 20 years of increasing old age, its impact on my mental well-being has been minimal.

I have never felt depressed, angry or upset by dysphagia itself, for I have been extremely fortunate over that time—having retired from my main job, moved into a beautiful village by the sea, and was living in a small convenient house within half a mile of my eldest son and his family, which gave me easy access to my son’s friends, contacts and local services when needed.

Additionally, my two other children live within a 90-min drive, I can enjoy the delights of plants and birds in my garden, I have had the loving companionship of an adorable dog for many years and have had wonderful support from an outstanding NHS hospital nearby. It has been an absolutely perfect situation in which to grow old!

With such extreme good fortune, feeling ‘lucky to be alive’ and still having many pressing interests in life, I have found it easy to keep the problems of dysphagia in proportion.

Finally, I cannot speak too highly of the support and services that I have always received from the NHS, but my thoughts in this paper are in fact a direct response to the views of Professor Smithard of the Lewisham and Greenwich NHS Trust, who believed that dysphagia had been a ‘Sleeping Giant’ within the NHS for many years [1].

I had never heard of the word ‘dysphagia’ until I began studying it on the Web early in 2020, and the word was never entered in my medical records. In fact, apart from two occasions, at my barium-swallow test in 2005 and on my request for a solution for my stringy saliva in 2009, I cannot remember my swallowing problems being mentioned or considered at any other visit to the NHS over many years.

All my regular visits to ENT between 2009 and 2018 included an inspection of my nose, throat and hearing, but were devoted to my balance problem and swallowing matters were not discussed.

There can surely be no doubt that further detailed medical research is desperately needed into how the detection, comprehensive diagnosis and dedicated clinical management of dysphagia could best be implemented and ensured across clinical services. Such research would include practical studies into the use of different liquids and the content and texture of different foods, to improve the rehabilitation of dysphagia worldwide.

## Data Availability

Not applicable.
